# Endocytosis of the thrombopoietin receptor Mpl regulates megakaryocyte and erythroid maturation in mice

**DOI:** 10.3389/fonc.2022.959806

**Published:** 2022-08-30

**Authors:** Nathan Eaton, Emily K. Boyd, Ratnashree Biswas, Melissa M. Lee-Sundlov, Theresa A. Dlugi, Haley E. Ramsey, Shikan Zheng, Robert T. Burns, Martha C. Sola-Visner, Karin M. Hoffmeister, Hervé Falet

**Affiliations:** ^1^ Translational Glycomics Center, Versiti Blood Research Institute, Milwaukee, WI, United States; ^2^ Department of Cell Biology, Neurobiology, and Anatomy, Medical College of Wisconsin, Milwaukee, WI, United States; ^3^ Division of Newborn Medicine, Boston Children’s Hospital, Boston, MA, United States; ^4^ Department of Pediatrics, Harvard Medical School, Boston, MA, United States; ^5^ Departments of Medicine and Biochemistry, Medical College of Wisconsin, Milwaukee, WI, United States

**Keywords:** mpl, dnm2, megakaryopoeiesis, erythropoiesis, hematopoiesis

## Abstract

*Dnm2^fl/fl^ Pf4-Cre* (*Dnm2^Plt–/–^*) mice lacking the endocytic GTPase dynamin 2 (DNM2) in platelets and megakaryocytes (MKs) develop hallmarks of myelofibrosis. At the cellular level, the tyrosine kinase JAK2 is constitutively active but decreased in expression in *Dnm2^Plt–/–^* platelets. Additionally, *Dnm2^Plt–/–^* platelets cannot endocytose the thrombopoietin (TPO) receptor Mpl, leading to elevated circulating TPO levels. Here, we assessed whether the hyperproliferative phenotype of *Dnm2^Plt–/–^* mice was due to JAK2 constitutive activation or to elevated circulating TPO levels. In unstimulated *Dnm2^Plt–/–^* platelets, STAT3 and, to a lower extent, STAT5 were phosphorylated, but their phosphorylation was slowed and diminished upon TPO stimulation. We further crossed *Dnm2^Plt–/–^* mice in the *Mpl^–/–^* background to generate *Mpl^–/–^Dnm2^Plt–/–^* mice lacking Mpl ubiquitously and DNM2 in platelets and MKs. *Mpl^–/–^ Dnm2^Plt–/–^* platelets had severely reduced JAK2 and STAT3 but normal STAT5 expression. *Mpl^–/–^ Dnm2^Plt–/–^* mice had severely reduced bone marrow MK and hematopoietic stem and progenitor cell numbers. Additionally, *Mpl^–/–^ Dnm2^Plt–/–^* mice had severe erythroblast (EB) maturation defects, decreased expression of hemoglobin and heme homeostasis genes and increased expression of ribosome biogenesis and protein translation genes in spleen EBs, and developed anemia with grossly elevated plasma erythropoietin (EPO) levels, leading to early fatality by postnatal day 25. *Mpl^–/–^ Dnm2^Plt+/+^* mice had impaired EB development at three weeks of age, which normalized with adulthood. Together, the data shows that DNM2-dependent Mpl-mediated endocytosis in platelets and MKs is required for steady-state hematopoiesis and provides novel insights into a developmentally controlled role for Mpl in normal erythropoiesis, regulating hemoglobin and heme production.

## Key Points

- Dynamin 2 (DNM2)-dependent Mpl-mediated endocytosis in platelets and megakaryocytes is required for steady-state hematopoiesis.
- Mpl developmentally regulates mouse erythropoiesis.

## Introduction

Blood platelets play an essential role in maintaining hemostasis and the integrity of the vasculature. High blood platelet count and platelet hyperactivation increase the risk of thrombosis and stroke, while low blood platelet count and platelet dysfunction predispose to hemorrhage. Platelets also participate in antimicrobial host defense and secrete cytokines that can induce inflammation and growth factors contributing to tissue repair. Thus, platelet homeostasis must be tightly regulated to avoid adverse effects of high or low platelet count.

Signaling of the hematopoietic cytokine thrombopoietin (TPO) through its receptor Mpl is essential for thrombopoiesis (1–3) and hematopoietic stem and progenitor cell (HSPC) maintenance (4–10). Patients with loss-of-function mutations in TPO or Mpl develop congenital amegakaryocytic thrombocytopenia (CAMT) and subsequent bone marrow failure (11–13). Mice lacking either TPO or Mpl have low megakaryocyte (MK) numbers and consequently develop severe thrombocytopenia (14–16). Hepatocytes are a major source of TPO, secreting the cytokine into the blood circulation (17). The mechanisms regulating circulating TPO levels are being debated. In one model, levels of circulating TPO are maintained solely by its uptake and metabolism by high-affinity Mpl receptors on platelets and MKs (18–23). In another model, circulating platelet levels regulate TPO mRNA expression in the liver by the proinflammatory cytokine interleukin 6 (IL-6), providing a regulated pathway to increase platelet production during acute inflammatory responses (24–26). More recent data suggest that the removal of aged, asialylated platelets stimulates hepatic TPO synthesis to maintain steady-state circulating TPO and platelet levels (27).

In platelets and MKs, the interaction between TPO and its receptor Mpl initiates an intracellular signaling cascade that involves the phosphorylation and activation of the tyrosine kinase JAK2 and the subsequent phosphorylation of signal transducer and activator of transcription (STAT) proteins (28). TPO binding to Mpl is also associated with cellular uptake of TPO and its subsequent degradation in a process regulated by receptor-mediated endocytosis (RME) (29, 30). RME plays an integral and physiologically relevant part in regulating plasma TPO levels. Cells expressing the JAK2V617F mutant commonly found in myeloproliferative neoplasm (MPN) patients display reduced recycling and increased degradation of Mpl, leading to elevated circulating TPO levels (31, 32). Mice specifically lacking Mpl or JAK2 in platelets and MKs, in which Mpl-mediated TPO endocytosis is blunted, display severe HSPC and MK hyperplasia and consequent thrombocytosis (33–35). However, the role of impaired TPO homeostasis in the HSPC and MK hyperplasia has not been conclusively demonstrated.

Dynamin 2 (DNM2) is a highly conserved GTPase essential for RME (36). *DNM2* mutations in humans have been associated with Charcot-Marie-Tooth disease, centronuclear myopathy, and early T-cell precursor acute lymphoblastic leukemia (37–39). Ubiquitous *Dnm2* deletion or loss of function in mice results in early embryonic lethality (40, 41). We have previously shown that *Dnm2^fl/fl^ Pf4-Cre* (*Dnm2^Plt–/–^*) mice lacking DNM2 in platelets and MKs develop HSPC and MK hyperplasia, extramedullary hematopoiesis, and splenomegaly (42). Additionally, *Dnm2^Plt–/–^* mice develop severe macrothrombocytopenia and bleeding (43). At the cellular level, *Dnm2^Plt–/–^* platelets display constitutive activation but decreased expression of JAK2 and are unable to endocytose Mpl, leading to elevated circulating TPO levels.

Here, we assessed whether the hyperproliferative phenotype of *Dnm2^Plt–/–^* mice was due to JAK2 constitutive activation or elevated circulating TPO levels. STAT3 and to a lower extent STAT5 were phosphorylated in unstimulated *Dnm2^Plt–/–^* platelets. However, their phosphorylation was slowed and diminished when *Dnm2^Plt–/–^* platelets were stimulated with TPO. Additional Mpl deletion resulted in the loss of JAK2 and STAT3, but not STAT5 in *Mpl^–/–^ Dnm2^Plt–/–^* platelets, linking JAK2 and STAT3 expression to Mpl and DNM2. At three weeks of age, *Mpl^–/–^ Dnm2^Plt–/–^* mice displayed a near complete depletion of bone marrow MKs and significantly reduced HSPCs, indicating that Mpl is the primary receptor contributing to the hyperproliferative phenotype of *Dnm2^Plt–/–^* mice. However, *Mpl^–/–^ Dnm2^Plt–/–^* mice showed severe anemia, erythroblast (EB) maturation defects, decreased expression of hemoglobin and heme homeostasis genes and increased expression of ribosome biogenesis and protein translation genes in spleen EBs, and grossly elevated plasma erythropoietin (EPO) levels, resulting in early fatality by postnatal day 25. *Mpl^–/–^ Dnm2^Plt+/+^* mice also displayed reduced EB development, which returned to normal with adulthood. Taken together, the data shows that DNM2-dependent Mpl-mediated endocytosis in platelets and MKs is required for steady-state hematopoiesis and provides novel insights into a developmentally controlled role for Mpl in normal erythropoiesis, regulating hemoglobin and heme production.

## Methods

### Mice

*Dnm2^Plt–/–^* mice were crossed with *Mpl^–/–^* mice to obtain mice lacking DNM2 in platelets and MKs and Mpl ubiquitously (14, 42). Mouse genotyping was confirmed by PCR of ear tissue DNA using primers: CCCTGCTAGTGACCTTTCTTGA (forward) and GCAGGAAGACACACAACTGAAC (reverse; *Dnm2^+^* 172bp and *Dnm2^fl^* 271bp); CCTGTATTCCCAGAGTGTGCC (forward), GGAGCTTGAGCAGGTAGAGAG (reverse; *Mpl^+^* 203bp), and CCAGCTCATTCCTCCCACTC (reverse; *Mpl^–^* 295bp); and AGATGCCAGGACATCAGGAACCTG (forward) and ATCAGCCACACCAGACACAGAGATC (reverse; *Pf4-iCre* 237bp). Mice were treated according to the National Institutes of Health and Medical College of Wisconsin Institutional Animal Care and Use Committee guidelines (Animal Use Application 5600).

### Platelet preparation

Mouse blood was collected from the retroorbital plexus in anticoagulant citrate dextrose solution (43). Platelet-rich plasma was obtained by centrifugation of the blood at 100 g for 8 min, followed by centrifugation of the supernatant and buffy coat at 100 g for 6 min. After washing twice in washing buffer (140 mM NaCl, 5 mM KCl, 12 mM trisodium citrate, 10 mM glucose, and 12.5 mM sucrose, pH 6.0), platelets were resuspended at 4 x 10 (8) platelets/ml in resuspension buffer (140 mM NaCl, 3 mM KCl, 0.5 mM MgCl2, 5 mM NaHCO3, 10 mM glucose, 10 mM HEPES, pH 7.4) and were allowed to rest for 30 min before use.

### Immunoblot analysis

Platelets were lysed in 1% Nonidet P-40, 150 mM NaCl, and 50 mM Tris/HCl, pH 7.4, containing 1 mM EGTA, 1 mM sodium orthovanadate, and cOmplete Protease Inhibitor Cocktail (Roche). SDS-PAGE buffer was added to lysates in the presence of 1% β-mercaptoethanol. Proteins were resolved by SDS-PAGE following quantification by Bradford protein assay and transferred onto PVDF membrane. After blocking overnight with 1% BSA in 0.2% Tween-20, 100 mM NaCl, and 20 mM Tris/HCl, pH 7.4, membranes were probed with rabbit antibodies directed against total or phosphorylated STAT3 (Tyr705) and STAT5 (Tyr694 in STAT5A; Tyr699 in STAT5B) (Cell Signaling), JAK2, β-actin, or GAPDH, followed by secondary horseradish peroxidase-conjugated goat anti-rabbit IgG antibody (Thermo Fisher Scientific). Detection was performed by enhanced chemiluminescence.

### Complete blood counts

Mouse blood was collected from retroorbital plexus and diluted in Cellpack (Sysmex) supplemented with EDTA and PGE_1_ (43, 44). Complete blood counts were measured on a Sysmex XT-2000i automatic hematology analyzer.

### TPO and EPO levels

Plasma EPO and TPO levels were quantified using a Mouse Thrombopoietin and Erythropoietin Quantikine ELISA kit (R&D Systems), respectively, following manufacturer recommendations (42).

### Cryosectioning and immunolabeling

Femurs of mice were fixed overnight in 1% paraformaldehyde/phosphate-lysine-sodium periodate and cryoprotected for at least 72 h in a 30% sucrose/phosphate buffer solution at 4°C before subsequent freezing in Sakura Tissue Tek O.C.T. compound (Andwin Scientific). Frozen cryosectioning on slides was performed at the Medical College of Wisconsin Histological Laboratory and Core Center. For MK counts, 7-µm femur sections were rehydrated and permeabilized in TBS-T for 15 min at room temperature (RT) then blocked overnight in a 5% BSA/PBS solution at 4°C. Sections were incubated for 2 h at RT with monoclonal rat-anti-GPIbα (Emfret Analytics) and polyclonal rabbit anti-laminin (Sigma-Aldrich) followed by a 1 h RT incubation with conjugated secondary antibodies (Molecular Probes). Sections were washed and mounted with Prolong Diamond Antifade Mountant with DAPI (Invitrogen) and imaged on a Nikon Eclipse Ti2-E platform equipped with a DS-Qi2 camera and Plan Apo 10x/0.45 (NIS-Elements AR 5.02.00 software). Data were image-processed using Imaris (Bitplane) and Matlab (Mathworks) softwares. Surfaces were created toward the greatest signal intensities GPIbα-positive cells and quantitatively analyzed using Imaris and Excel (Microsoft).

### Bone marrow and spleen histology

Mouse femurs and spleens were fixed overnight in 4% paraformaldehyde/PBS. Bones were decalcified in 0.5 M EDTA, pH 8.0 (Boston BioProducts) for 7 days under rotation, exchanging EDTA twice daily. Tissues were paraffin embedded, and sections were stained with hematoxylin and eosin (H&E) at the Versiti Blood Research Institute and Medical College of Wisconsin Histology Core.

### Blood smears

Blood smears were performed *via* Wright-Giemsa stain. Anticoagulated whole blood was thinly smeared across a glass slide and fixed for 3 min in methanol, stained 1 min in Wright-Giemsa, and washed for 5 min in PBS. Imaging was performed on a Nikon Eclipse E600 microscope equipped with a SPOT insight firewire color mosaic camera (SPOT imaging solutions) and Plan Apo 40x/0.75 objective, with SPOT imaging 5.1.3 software.

### Flow cytometry analysis

Spleen and bone marrow cells were collected and homogenized through a 70-µm filter. For the EB analysis, spleen cells were stained with FITC-conjugated anti-CD71 and PE-conjugated anti-TER-119 (BD Biosciences) after homogenization and washing (45, 46). For the HSPC analysis, bone marrow cells were prepared for staining by erythrocyte lysis (BD Pharm Lyse; BD Biosciences). Cells were then stained in ice-cold PBS containing 2% FBS using the following antibodies: lineage cocktail containing TER-119, CD11b (Mac-1), Ly-6G/Ly-6C (Gr-1), CD3ϵ, and CD45R (B220); CD117 (Kit); Ly-6A/E (Sca-1); CD150 (Slamf1); CD48 (Slamf2); CD16/32; CD34; CD41 (Itga2b); and CD105 (endoglin) (BioLegend and eBiosciences). 4′,6 Diamidino-2-phenylindole (Invitrogen) was used for dead cell discrimination. SLAM and MKEP panels used were described previously (47–49). Samples were analyzed by flow cytometry using an LSR II (BD Biosciences). Post-acquisition analysis of data was performed with FlowJo software.

### Erythroblast *Dnm2* DNA analysis

Spleen cells were collected and homogenized through a 70-µm filter and leukocytes were depleted using anti-CD45 beads (Miltenyi Biotec). Remaining cells were stained with FITC-conjugated anti-CD71, PE-conjugated anti-TER-119, and APC-conjugated anti-CD45 as control. Immature CD71^high^ EBs were collected on a FACSAria II cell sorter (BD Biosciences). EB genomic DNA was obtained using the QIAamp Mini DNA kit (Qiagen). Duplicates of real-time PCR experiments were performed on a QuantStudio 6 Flex Real-Time PCR System (Applied Biosystems) amplifying *Dnm2* and *Rn18s* as reference (50). Primers used were: CCCTGCTAGTGACCTTTCTTGA (forward) and GCAGGAAGACACACAACTGAAC (reverse; *Dnm2^fl^* 271bp); and TTGACGGAAGGGCACCACCAG (forward) and GCACCACCACCCACGGAATCG (reverse; *Rn18s* 131bp). Ct numbers were extracted for both *Dnm2* and *Rn18s* with auto baseline and manual threshold.

### Erythroblast Bulk RNA sequencing analysis

EB RNA was isolated using Trizol reagent (Invitrogen) and an Autogen Prep-245 system and was assessed with the Bioanalyzer RNA Nano Assay (Agilent). All samples had observed RNA Integrity Number values >7.4 with DV200 over 81%. RNA libraries were prepared (Illumina TruSeq Stranded mRNA, single indexed) and run on the Illumina High Seq-2500 for 125bp paired end reads at the Medical College of Wisconsin Genomic Sciences and Precision Medicine Center. Samples were sequenced to an average depth of 40 million reads. All data was quality controlled using FastQC and RSeQC, followed by manual review and data visualization (51). Bulk RNA-seq data were aligned to the Mus musculus mm10 genome and quality control was performed using Nextflow pipeline (nf-core/rnaseq 1.4.2) (DOI:10.5281/zenodo.1400710) (52). Gene expression was quantified at the gene level using Salmon. RNA-seq libraries were then normalized and genes were tested for differential expression between *Dnm2^Plt+/+^*, *Dnm2^Plt–/–^*, *Mpl^–/–^ Dnm2^Plt+/+^*, and *Mpl^–/–^ Dnm2^Plt–/–^* samples with DESeq2 v1.24.0 (53). DESeq2 Wald tests were used to determine whether fold changes were significantly different from zero. For visualization, data were transformed using the regularized logarithm transformation (53). Pre-ranked gene set enrichment analyses were conducted using shrunken fold-changes and clusterProfiler v3.12.0 (54). Kyoto Encyclopedia of Genes and Genomes (KEGG), Reactome, and GO databases were used for Gene Set Enrichment Analysis (GSEA) (55–57). The Benjamini-Hochberg method was used to adjust p-values for false-discovery in both differential expression and GSEA analyses (58). Genes were defined as differentially expressed if they were upregulated or downregulated 1.5-fold with an adjusted *P*-value <.05.

### Statistical analysis

Results were compared statistically with the unpaired Student’s t-test (mean comparison between two groups), one- and two-way ANOVA (mean comparison between multiple groups), and the Log-rank test (survival distribution comparison) using Prism software (GraphPad). Differences were considered statistically significant when *P* <.05.

## Results

### Impaired STAT signaling in *Dnm2^Plt–/–^* platelets

**Figure 1:**
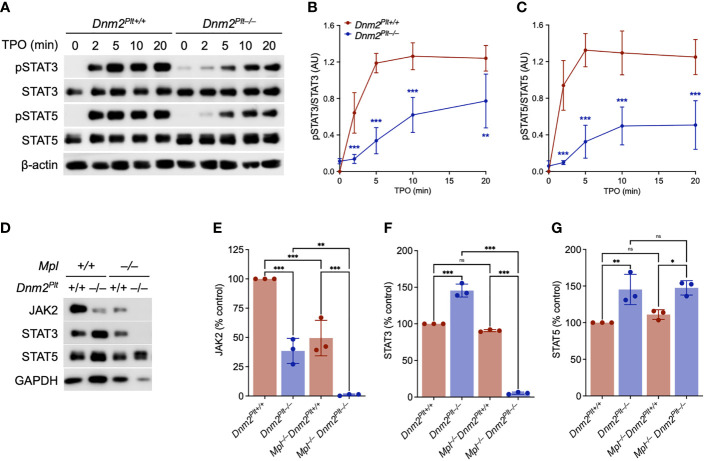
JAK2-STAT signaling defects in *Dnm2^Plt–/–^* platelets. **(A)** *Dnm2^Plt+/+^* and *Dnm2^Plt–/–^* platelets were activated with 50 ng/ml TPO for 10 min at 37°C, lysed, subjected to SDS-PAGE, and probed for total and phosphorylated STAT3 (pSTAT3; Tyr705), total and phosphorylated STAT5 (pSTAT5; Tyr694 in STAT5A, Tyr699 in STAT5B), and β-actin as loading control, as indicated. Densitometry analysis of STAT3 **(B)** and STAT5 **(C)** phosphorylation. Results represent mean ± SD of 4 independent experiments and are compared statistically by two-way ANOVA (**,*P* <.01; ***, *P* <.001). **(D)** Platelet lysates of *Dnm2^Plt+/+^*, *Dnm2^Plt–/–^*, *Mpl^–/–^ Dnm2^Plt+/+^*, and *Mpl^–/–^ Dnm2^Plt–/–^* mice at P24 corresponding to 2 µg of protein were subjected to SDS-PAGE and probed for JAK2, STAT3, STAT5, and GAPDH as loading control, as indicated. Results are representative of 3 independent experiments. Densitometry analysis of JAK2 **(E)**, STAT3 **(F)**, and STAT5 **(G)** expression. Results represent mean ± SD of 3 independent experiments and are compared statistically by one-way ANOVA (ns, not significant, *P <.05; **P <.01; ***P <.001).

The TPO-Mpl interaction initiates a signaling cascade that involves JAK2 activation and the subsequent phosphorylation of STAT proteins (28). *Dnm2^Plt–/–^* mice specifically lacking DNM2 in platelets and MKs develop HSPC and MK hyperplasia, extramedullary hematopoiesis, and splenomegaly (42). While JAK2 expression is decreased in *Dnm2^Plt–/–^* platelets, its TPO-independent phosphorylation at tyrosine residues 1007 and 1008 indicates constitutive activation (42). To understand the alterations in JAK2-STAT signaling, we evaluated the phosphorylation of STAT3 and STAT5 in *Dnm2^Plt–/–^* platelets (**Figures 1A−C**). As expected, STAT3 and STAT5 were not phosphorylated in unstimulated control *Dnm2^Plt+/+^* platelets. Incubation of *Dnm2^Plt+/+^* platelets with 50 ng/ml of TPO resulted in STAT3 and STAT5 phosphorylation on tyrosine residues 705 and 694/699, respectively, that began at 2 min and became maximal at 5 min. We observed STAT3 and to a lower extent STAT5 phosphorylation in unstimulated *Dnm2^Plt–/–^* platelets. However, compared to controls, STAT3 and STAT5 phosphorylation was slowed and diminished in *Dnm2^Plt–/–^* platelets following stimulation with TPO. The data is consistent with JAK2 constitutive activation but decreased expression in *Dnm2^Plt–/–^* platelets.

To ascertain the role of impaired Mpl-mediated endocytosis in the hyperproliferative phenotype, we crossed *Dnm2^Plt–/–^* mice in the *Mpl^–/–^* background to generate mice lacking DNM2 in platelets and MKs and Mpl ubiquitously. We measured a ~60% and ~50% reduction in JAK2 expression in platelets lacking DNM2 and Mpl, respectively (**Figures 1D, E**). By contrast, expression of STAT3 and STAT5 was increased by ~40% in platelets lacking DNM2, but was unaffected by Mpl deletion (**Figures 1D, F, G**). The combined deletion of DNM2 and Mpl resulted in the loss of JAK2 and STAT3, but not STAT5 in *Mpl^–/–^ Dnm2^Plt–/–^* platelets (**Figures 1D−G**). Together, the data links JAK2 and STAT3, but not STAT5 homeostasis in platelets to Mpl and DNM2 expression. While proteins were loaded according to protein amount, no standard controls (β-actin, β-tubulin, GAPDH) gave a good signal for *Mpl^–/–^ Dnm2^Plt–/–^* platelets, suggesting major protein up- and down-regulation.

### Early lethality in *Mpl^–/–^ Dnm2^Plt–/–^* mice

**Figure 2:**
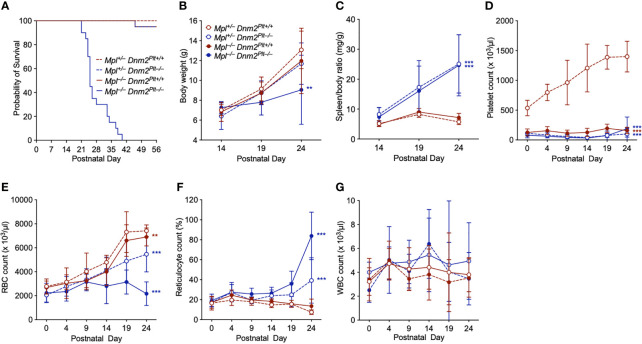
*Mpl^–/–^ Dnm2^Plt–/–^* mice exhibit early lethality, failure to thrive, splenomegaly, and severe anemia. **(A)** Survival of *Mpl^+/–^ Dnm2^Plt+/+^*, *Mpl^+/–^ Dnm2^Plt–/–^*, *Mpl^–/–^ Dnm2^Plt+/+^*, and *Mpl^–/–^ Dnm2^Plt–/–^* littermates. Results are estimated using the Kaplan-Meier method and are compared statistically using the Log-rank test (n = 20 mice in each group: ***, Log-rank *P* <.001). Body weights **(B)** and spleen/body ratio **(C)** at P14, P19, and P24. Platelet **(D)**, RBC **(E)**, reticulocyte **(F)**, and WBC **(G)** counts from birth (P0) to postnatal day 24 (P24). Results represent mean ± SD of 5-19 independent experiments and are compared statistically by two-way ANOVA (**P <.01; ***P <.001).

We crossed *Mpl^–/–^ Dnm2^Plt+/+^* and *Mpl^+/–^ Dnm2^Plt–/–^* mice and obtained four offspring genotypes, i.e., *Mpl^+/–^ Dnm2^Plt+/+^*, *Mpl^+/–^ Dnm2^Plt–/–^*, *Mpl^–/–^ Dnm2^Plt+/+^*, and *Mpl^–/–^ Dnm2^Plt–/–^*, with a normal Mendelian inheritance ratio at birth (data not shown). *Mpl^+/–^ Dnm2^Plt+/+^*, *Mpl^+/–^ Dnm2^Plt–/–^*, and *Mpl^–/–^ Dnm2^Plt+/+^* mice reached adulthood (**Figure 2A**), as described previously for *Dnm2^Plt–/–^* and *Mpl^–/–^* mice (14, 42). By contrast, *Mpl^–/–^ Dnm2^Plt–/–^* mice became pale and moribund (data not shown) and died at a median age of 25 days postnatally (Log-rank *P* <.001).

We measured body weights at postnatal day 14 (P14), P19, and P24 (**Figure 2B**). While *Mpl^+/–^ Dnm2^Plt+/+^*, *Mpl^+/–^ Dnm2^Plt–/–^*, and *Mpl^–/–^ Dnm2^Plt+/+^* mice gained weight over time, *Mpl^–/–^ Dnm2^Plt–/–^* mice failed to thrive after P14 and showed diminutive growth at P24, with a body weight of 9.05 ± 3.48 g, compared to 13.07 ± 1.88 g in *Mpl^+/–^ Dnm2^Plt+/+^* littermates, a 31% decrease (*P* = .005). DNM2 deletion in platelets and MKs led to severe splenomegaly, independently of Mpl expression (**Figure 2C**), indicating that the extramedullary hematopoiesis of *Dnm2^Plt–/–^* mice was not related to impaired Mpl-mediated endocytosis in platelets and MKs.

### Severe anemia in *Mpl^–/–^ Dnm2^Plt–/–^* mice

To understand the cause of early mortality of *Mpl^–/–^ Dnm2^Plt–/–^* mice, we measured hematological parameters between birth (P0) and P24. In *Mpl^+/–^ Dnm2^Plt+/+^* mice, the platelet count increased from 536 ± 130 x 10^3^/µl at P0 to 1398 ± 257 x 10^3^/µl at P24 (**Figure 2D**), as described for control *Mpl^+/+^* mice (44). Consistent with previous observations (14, 42), mice lacking DNM2 in platelets and MKs and/or Mpl ubiquitously developed severe thrombocytopenia, with platelet counts constantly below 200 x 10^3^/µl, which was observed at birth and throughout development.

The RBC count rose continuously in *Mpl^+/–^ Dnm2^Plt+/+^*, *Mpl^+/–^ Dnm2^Plt–/–^*, and *Mpl^–/–^ Dnm2^Plt+/+^* mice (**Figure 2E**). By contrast, the RBC count failed to increase after P14 in *Mpl^–/–^ Dnm2^Plt–/–^* mice, which displayed severe anemia and an aberrant increase in reticulocyte count at P24 (**Figure 2F**). All four mouse genotypes had a normal white blood cell (WBC) count throughout development (**Figure 2G**). Together, the data suggested that DNM2 deletion in platelets and MKs combined with Mpl ubiquitous deletion induced an age-dependent lethal anemia in *Mpl^–/–^ Dnm2^Plt–/–^* mice. We, therefore, investigated megakaryopoiesis and erythropoiesis in further detail.

### The MK hyperplasia of *Dnm2^Plt–/–^* mice requires Mpl expression

**Figure 3:**
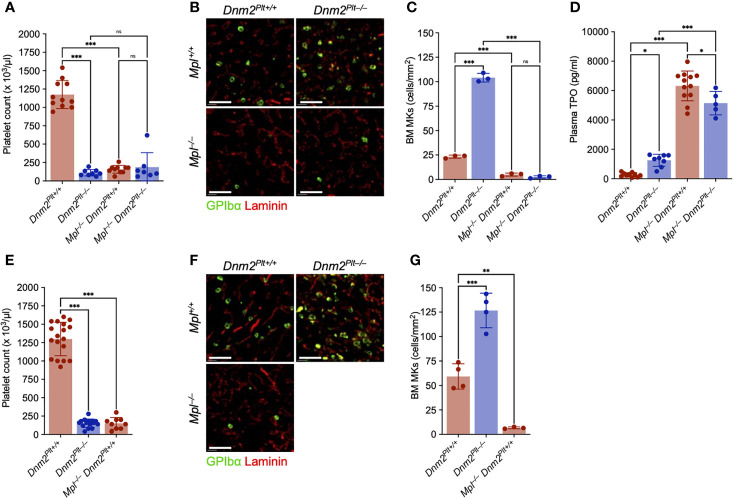
The MK hyperplasia of *Dnm2^Plt–/–^* mice requires Mpl expression. Platelet count at P24 **(A)** and P56 **(E)**. Results represent mean ± SD of 7-13 independent experiments and are compared statistically by one-way ANOVA (ns, not significant, ***, *P* <.001). Seven-µm frozen bone marrow sections of *Dnm2^Plt+/+^*, *Dnm2^Plt–/–^*, *Mpl^–/–^ Dnm2^Plt+/+^*, and *Mpl^–/–^ Dnm2^Plt–/–^* mice at P24 **(B)** and P56 **(F)** were probed for resident MKs (GPIbα, green) and bone marrow vasculature (laminin, red). Sections shown are representative of 3-4 mice in each genotype. Scale bars represent 150 µm. Bone marrow MK numbers at P24 **(C)** and P56 **(G)**. Results represent mean ± SD of 3-4 independent experiments and are compared statistically by one-way ANOVA (ns, not significant, **P <.01; ***P <.001). **(D)** Plasma TPO levels at P24. Results represent mean ± SD of 5-16 independent experiments and are compared statistically by one-way ANOVA (*P <.05; **P <.01; ***P <.001).

We evaluated platelet counts and bone marrow megakaryopoiesis at P24 and P56 (**Figure 3**). At P24, DNM2 deletion in platelets and MKs and/or Mpl ubiquitous deletion resulted in comparable severe thrombocytopenia with platelet counts of 111 ± 43 x 10^3^/µl in *Dnm2^Plt–/–^*, 158 ± 53 x 10^3^/µl in *Mpl^–/–^ Dnm2^Plt+/+^*, and 189 ± 196 x 10^3^/µl in *Mpl^–/–^ Dnm2^Plt–/–^*, compared to 1243 ± 300 x 10^3^/µl in control *Dnm2^Plt+/+^* mice (*P* <.0001) (**Figure 3A**). Similar low platelet counts were obtained at P56, with 152 ± 57 x 10^3^/µl in *Dnm2^Plt–/–^* and 151 ± 79 10^3^/µl in *Mpl^–/–^ Dnm2^Plt+/+^* mice, compared to 1299 ± 227 x 10^3^/µl in control *Dnm2^Plt+/+^* mice (*P* <.001) (**Figure 3E**). Due to their early lethality, hematological parameters could not be evaluated at P56 for *Mpl^–/–^ Dnm2^Plt–/–^* mice.

We evaluated bone marrow MKs by immunofluorescence microscopy using an antibody directed against GPIbα. At P24, *Dnm2^Plt–/–^* mice displayed a severe MK hyperplasia, with 104.1 ± 4.5 MKs/mm^2^, compared to 23.3 ± 1.6 MKs/mm^2^ in control *Dnm2^Plt+/+^* mice (*P* <.001), a 4.5-fold increase (**Figures 3B**, **C**). Mpl deletion resulted in near-complete depletion of bone marrow MKs in *Mpl^–/–^ Dnm2^Plt–/–^* mice (2.5 ± 1.3 MKs/mm^2^), like *Mpl^–/–^ Dnm2^Plt+/+^* mice (5.0 ± 1.6 MKs/mm^2^). Comparable results were obtained by H&E staining in femur bone marrow and spleen tissues (**Supplementary Figures 1A, B**), and the MK hypoplasia of *Mpl^–/–^ Dnm2^Plt+/+^* mice remained at P56 (**Figures 3F, G**). The data demonstrated that the MK hyperplasia of *Dnm2^Plt–/–^* mice required the expression of the TPO receptor Mpl.

Consistent with the loss of DNM2-dependent Mpl-mediated endocytosis, plasma TPO levels were elevated in mice lacking DNM2 in platelets and MKs and/or Mpl ubiquitously: 1253 ± 411 pg/ml in *Dnm2^Plt–/–^*, 6318 ± 1014 pg/ml in *Mpl^–/–^ Dnm2^Plt+/+^*, and 5144 ± 800 pg/ml in *Mpl^–/–^ Dnm2^Plt–/–^*, compared to 269 ± 139 pg/ml in control *Dnm2^Plt+/+^* mice (*P* <.001) (**Figure 3D**). Together, the data showed that Mpl-mediated endocytosis in platelets and MKs required DNM2 expression to regulate plasma TPO levels.

### Severe erythroid maturation defects in *Mpl^–/–^ Dnm2^Plt–/–^* mice

**Figure 4:**
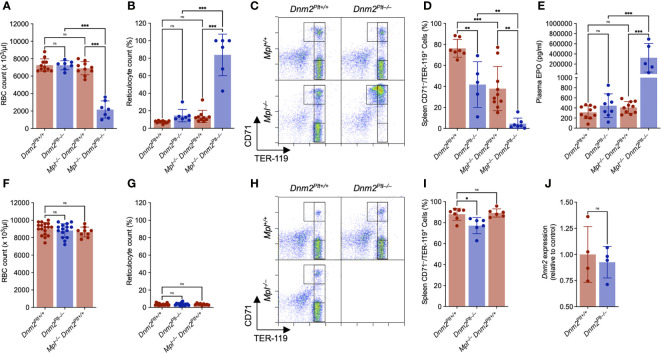
*Mpl^–/–^ Dnm2^Plt–/–^* mice exhibit impaired erythroid development at P24. RBC count at P24 **(A)** and P56 **(F)**. Reticulocyte count at P24 **(B)** and P56 **(G)**. Results represent mean ± SD of 4-18 independent experiments and are compared statistically by one-way ANOVA (ns, not significant, ***P <.001). Flow cytometry profiles of spleen EBs at P24 **(C)** and P56 **(H)** using the erythroid markers CD71 and TER-119. Data shown are representative of 3-13 mice in each genotype. Percentage of mature spleen CD71^low^/TER-119^high^ EBs at P24 **(D)** and P56 **(I)**. Results represent mean ± SD of 5-10 independent experiments and are compared statistically by one-way ANOVA (ns, not significant, *P <.05; **P <.01; ***P <.001). Plasma EPO levels at P24 **(E)**. Results represent mean ± SD of 5-15 independent experiments and are compared statistically by one-way ANOVA (ns, not significant, ***P <.001). **(J)** Genomic *Dnm2* expression in isolated spleen CD71^high^ EBs was evaluated by qPCR and normalized to *Dnm2^Plt+/+^* cells. Results represent mean ± SD of 4 independent experiments and are compared statistically by Student’s t-test. (ns, not significant).

Because of the escalating RBC deficit and death at P25, we evaluated RBC counts and erythroid maturation more in detail at P24 (**Figures 4A−E**). *Dnm2^Plt–/–^* and *Mpl^–/–^ Dnm2^Plt+/+^* mice had normal RBC counts with and 7247 ± 513 x 10^3^/µl and 6900 ± 750 x 10^3^/µl, respectively, compared to 7255 ± 727 x 10^3^/µl in control *Dnm2^Plt+/+^* mice (**Figure 4A**), indicating that individual loss of either DNM2 in platelets and MKs or Mpl ubiquitously does not affect RBC counts. By contrast, DNM2 deletion in platelets and MKs combined with ubiquitous Mpl deletion led to a significant decrease in RBC count to 2171 ± 983 x 10^3^/µl in *Mpl^–/–^ Dnm2^Plt–/–^* mice (*P* <.001). The severe anemia of *Mpl^–/–^ Dnm2^Plt–/–^* mice was accompanied by a grossly elevated reticulocyte count of 83.8 ± 23.8%, compared to 7.3 ± 1.4%, 14.6 ± 7.1%, and 13.5 ± 7.0% in *Dnm2^Plt+/+^*, *Dnm2^Plt–/–^*, and *Mpl^–/–^ Dnm2^Plt+/+^* mice, respectively (*P* <.001) (**Figure 4B**). Analyzing thin blood smears showed that *Mpl^–/–^ Dnm2^Plt–/–^* mice developed marked polychromasia (**Supplementary Figure 1C**), suggesting premature release during RBC formation.

Erythroid maturation at P24 was evaluated by flow cytometry analysis using CD71 and TER-119 as surface erythroid markers (**Figure 4C**), where immature EBs are defined as CD71^high^/TER-119^low^ and mature EBs as CD71^low^/TER-119^high^ (45, 46). In the spleens of control *Dnm2^Plt+/+^* mice, 76.3 ± 8.5% of erythroid cells were mature CD71^low^/TER-119^high^ EBs (**Figure 4D**). The population decreased to 41.7 ± 21.7% in *Dnm2^Plt–/–^* mice (*P* = .006) and 37.9 ± 21.1% in *Mpl^–/–^ Dnm2^Plt+/+^* mice (*P* <.001). In the spleens of *Mpl^–/–^ Dnm2^Plt–/–^* mice, only 4.5 ± 5.3% (*P* <.001) of erythroid cells were marked as mature EBs, as the majority failed to properly develop beyond an earlier CD71^high^ stage.

While plasma EPO levels were in the normal range in *Dnm2^Plt–/–^* and *Mpl^–/–^ Dnm2^Plt+/+^* mice, they were grossly elevated in *Mpl^–/–^ Dnm2^Plt–/–^* mice, with 325 ± 282 ng/ml, compared to 314 ± 128 pg/ml in control *Dnm2^Plt+/+^* mice (*P* <.001), a ~1000-fold increase (**Figure 4E**). The data showed that the severe anemia of *Mpl^–/–^ Dnm2^Plt–/–^* mice was due to a blockade of erythroid maturation at an early CD71^high^ stage. Thus, the loss of DNM2 in platelets and MKs combined with Mpl ubiquitous deletion caused a severe defect in erythrocyte development and maturation.

### Mpl contributes to erythroblast maturation during early mouse development

While Mpl deficiency has been associated with pancytopenia in humans (59), anemia has not been observed in mice lacking either Mpl or TPO, which mainly develop thrombocytopenia (14–16). To understand how our observations at P24 differ from these previous studies, we evaluated RBC counts and erythroid maturation in adult mice (**Figures 4F−I**). As observed at P24, the RBC and reticulocyte count at P56 was normal in *Dnm2^Plt–/–^* and *Mpl^–/–^ Dnm2^Plt+/+^* mice (**Figures 4F, G**). Evaluating erythroid maturation further, the mature CD71^low^/TER-119^high^ EB population in *Dnm2^Plt–/–^* mice increased between P24 and P56 to 77.2 ± 7.8%, although it did not reach control levels (*P* = .01) (**Figures 4H, I**). *Mpl^–/–^ Dnm2^Plt+/+^* mice had normal erythropoiesis at P56, with 89.3 ± 3.9% mature EBs, compared to 88.1 ± 5.7% in control *Dnm2^Plt+/+^* mice. Thus, the TPO-Mpl dependence of erythroid maturation was not evident when mice reached adulthood, demonstrating a developmental role for Mpl in erythropoiesis.

### DNM2 is normally expressed in *Dnm2^Plt–/–^* erythroblasts

Because ubiquitous *Dnm2* deletion or loss of function leads to microcytic anemia and embryonic lethality (40, 41), we investigated whether *Dnm2* was erroneously excised in *Dnm2^Plt–/–^* EBs (**Figure 4J**). Genomic DNA was collected from *Dnm2^Plt+/+^* and *Dnm2^Plt–/–^* CD71^high^ spleen EBs and probed by quantitative PCR using primers flanking the 5’ *Dnm2* loxP site, in conditions where the reverse *Dnm2* primer would not have a template to anneal to and no PCR product would be generated if the region between the two *Dnm2* loxP sites had been excised (40). *Dnm2^Plt+/+^* and *Dnm2^Plt–/–^* EBs yielded comparable amount of *Dnm2^fl^* PCR product, compared to control 18S rRNA, demonstrating that *Dnm2* was not excised and therefore DNM2 was normally expressed in *Dnm2^Plt–/–^* EBs.

### Hematopoietic stem and progenitor cell (HSPC) dysregulation in *Mpl^–/–^ Dnm2^Plt–/–^* mice

**Figure 5:**
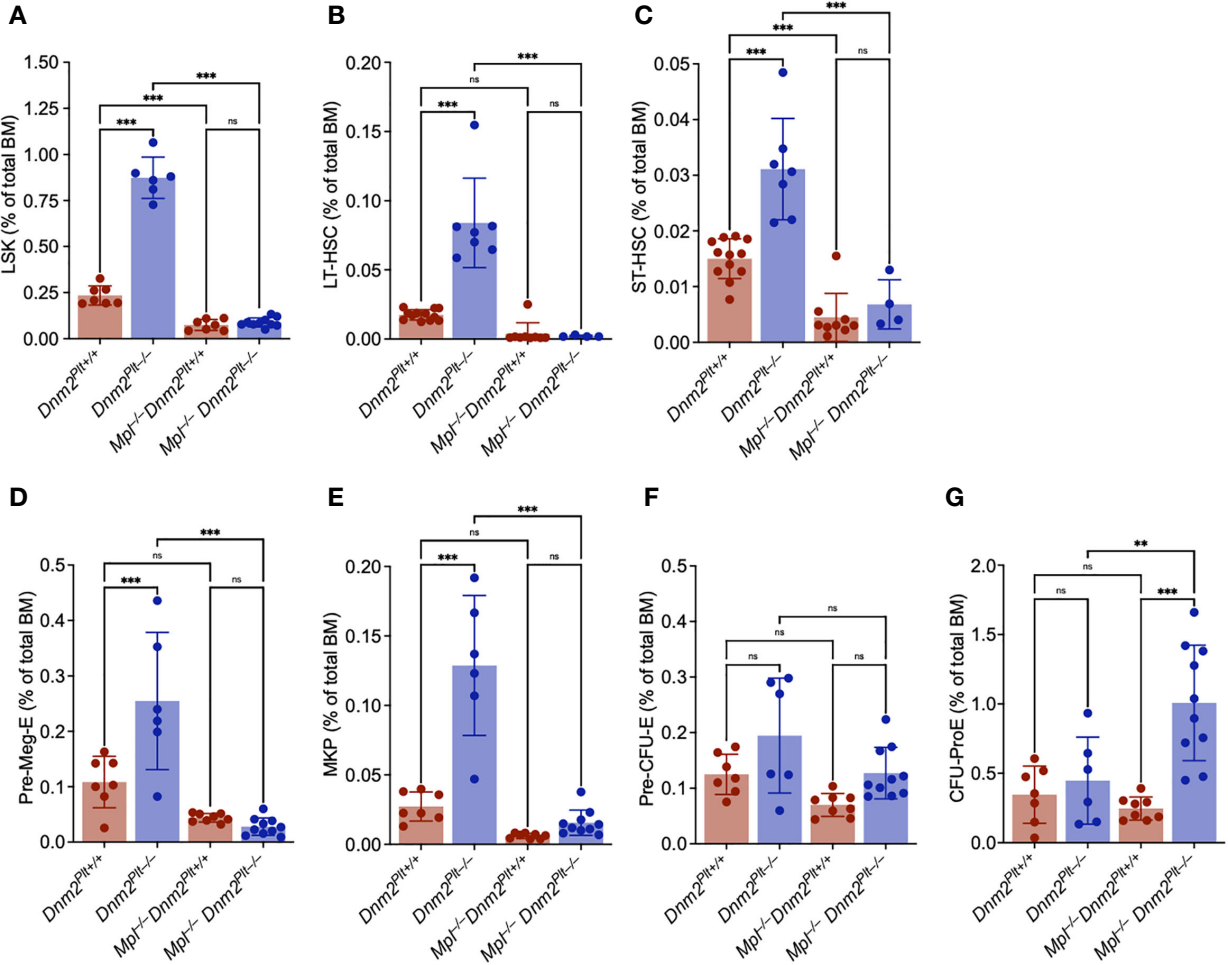
The increased HSPC expansion of *Dnm2^Plt–/–^* mice requires Mpl expression. Frequency of bone marrow Lin^–^/Sca1^+^/Kit^+^ (LSK) **(A)**, long-term (LT)-HSC **(B)**, short-term (ST)-HSC **(C)**, Pre-Meg-E **(D)**, MK progenitor (MKP) **(E)**, Pre-CFU-E **(F)**, and CFU-ProE **(G)** in *Dnm2^Plt+/+^*, *Dnm2^Plt–/–^*, *Mpl^–/–^ Dnm2^Plt+/+^*, and *Mpl^–/–^ Dnm2^Plt–/–^* mice at P24. Results represent mean ± SD of 4-12 independent experiments and are compared statistically by one-way ANOVA (**P <.01; ***P <.001). ns, not significant.

We performed a quantitative assessment of the bone marrow HSPC compartment at P24 using flow cytometry and well-established immunophenotypic markers (**Figure 5**) (47–49). Compared to control *Dnm2^Plt+/+^* mice, the lineage^–^/Sca-1^+^/Kit^+^ (LSK) compartment of *Dnm2^Plt–/–^* mice was significantly expanded (**Figure 5A**), including long-term CD150^+^/CD48^–^ (**Figure 5B**) and short-term CD150^–^/CD48^–^ (**Figure 5C**) HSCs (LT- and ST-HSCs, respectively). The LSK compartment was severely and equally reduced in *Mpl^–/–^ Dnm2^Plt+/+^* and *Mpl^–/–^ Dnm2^Plt–/–^* mice. The lack of LT- and ST-HSCs in the absence of Mpl is consistent with the reported requirement for TPO and Mpl in regulating HSCs (15, 60, 61).

MK and erythroid progenitors were evaluated in further details. Consistent with the observed MK hyperplasia and studies in adult mice (42), *Dnm2^Plt–/–^* mice displayed an expansion of the Pre-Meg-E (**Figure 5D**) and MK progenitor (MKP) (**Figure 5E**) compartments. The Pre-Meg-E and MKP expansion was abrogated in *Mpl^–/–^ Dnm2^Plt–/–^* mice, demonstrating that it was mediated by Mpl ubiquitous expression. While the Pre-CFU-E compartment was minimally affected by deletion of either DNM2 in platelets and MKs or Mpl ubiquitously (**Figure 5F**), the CFU-ProE compartment was elevated in *Mpl^–/–^ Dnm2^Plt–/–^* mice (**Figure 5G**). Together, the data demonstrated that the loss of DNM2-dependent Mpl-mediated endocytosis in platelets and MKs was responsible for the expansion of the LSK, Pre-Meg-E, and MKP compartments in *Dnm2^Plt–/–^* mice. It also led to an expansion of the CFU-ProE compartment in *Mpl^–/–^ Dnm2^Plt–/–^* mice, consistent with the observed erythroid maturation arrest at the stage of CD71^high^ immature EBs.

### Genome-wide transcriptome effects in erythroblasts

To evaluate the role of Mpl ubiquitous deletion and DNM2 deletion in platelets and MKs on erythropoiesis, spleen EBs isolated from *Dnm2^Plt+/+^*, *Dnm2^Plt–/–^*, *Mpl^–/–^ Dnm2^Plt+/+^*, and *Mpl^–/–^ Dnm2^Plt–/–^* mice at P24 were subjected to transcriptional profiling (n = 3 in each cohort). A total of 35,324 genes were retained, and two separate gene sets were generated to assess the effects of Mpl ubiquitous deletion or DNM2 deletion in platelets and MKs.

**Figure 6:**
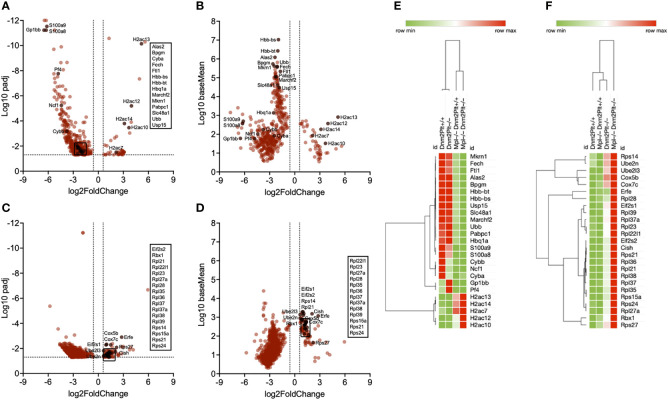
Transcriptional profiling of spleen EBs in *Dnm2^Plt+/+^*, *Dnm2^Plt–/–^*, *Mpl^–/–^ Dnm2^Plt+/+^*, and *Mpl^–/–^ Dnm2^Plt–/–^* mice. Volcano plots showing genes differentially expressed in EBs of *Mpl^–/–^ Dnm2^Plt+/+^* + *Mpl^–/–^ Dnm2^Plt–/–^* versus *Dnm2^Plt+/+^* + *Dnm2^Plt–/–^* mice (Mpl effect), displayed as adjusted *P*-value **(A)** and baseMean **(B)**. Volcano plots showing genes differentially expressed in EBs of *Dnm2^Plt–/–^* + *Mpl^–/–^ Dnm2^Plt–/–^* versus *Dnm2^Plt+/+^* + *Mpl^–/–^ Dnm2^Plt+/+^* mice (DNM2 effect), displayed as adjusted *P*-value **(C)** and baseMean **(D)**. The log2FoldChange indicates the mean expression level change for each gene. Dashed lines indicate fold changes of -1.5 and 1.5 (x-axis) and adjusted *P*-value of.05 (y-axis). Each dot denotes one gene. Heat maps showing relevant genes identified using the Mpl **(E)** and DNM2 **(F)** effects in all four groups.

Following the analysis of *Mpl^–/–^ Dnm2^Plt+/+^* and *Mpl^–/–^ Dnm2^Plt–/–^*, compared to *Dnm2^Plt+/+^* and *Dnm2^Plt–/–^* mice, the Mpl effect gene set contained 45 upregulated and 401 downregulated genes (**Figures 6A, B**; **Supplementary Table 1**). Notably, hemoglobin (Hbb-bs, Hbb-bt, Hbq1a) and heme homeostasis genes (Alas2, Fech, Bpgm, Ftl1, Slc48a1) were downregulated. Delving further into the transcriptomic data, the heat map of average FPKM values from all four groups showed that hemoglobin and heme homeostasis genes were severely decreased in mice lacking Mpl ubiquitously, independently of DNM2 deletion in platelets and MKs (**Figure 6E**). Together, the data indicate that Mpl plays a critical role in EB maturation by regulating hemoglobin and heme production, both critical to erythropoiesis (62). Additional relevant downregulated genes are involved in ubiquitination (Ubb, Mkrn1, Marchf2, Usp15) and ribosome recruitment and translation initiation (Pabpc1). Alternatively, H2A cluster histone genes (H2ac7, H2ac10, H2ac12, H2ac13, H2ac14) were upregulated in *Mpl^–/–^ >Dnm2^Plt+/+^* and *Mpl^–/–^Dnm2^Plt–/–^* EBs, compared to *Dnm2^Plt+/+^* and *Dnm2^Plt–/–^* EBs. Genes involved in inflammation (S100a8, S100a9, Cyba, Cybb, Ncf1) were decreased in all three thrombocytopenic genotypes, while platelet genes (Gp1bb, Pf4) were elevated in *Dnm2^Plt–/–^* mice presenting MK hyperplasia (**Figure 6E**).

Following the analysis of *Dnm2^Plt–/–^* and *Mpl^–/–^ Dnm2^Plt–/–^*, compared to *Dnm2^Plt+/+^* and *Mpl^–/–^ Dnm2^Plt+/+^* mice, the DNM2 effect gene set contained 89 upregulated and 803 downregulated genes (**Figures 6C, D**; **Supplementary Table 2**). E2 ubiquitin-conjugating enzymes (Ube2l3, Ube2n) and cytochrome c oxidase genes (Cox5b, Cox7c) were upregulated in mice lacking DNM2 in platelets and MKs, independently of Mpl ubiquitous deletion (**Figure 6F**). E3 ubiquitin ligase genes (Cish, Rbx1), genes involved in ribosome biogenesis (Rpl21, Rpl22l1, Rpl23, Rpl27a, Rpl28, Rpl35, Rpl36, Rpl37, Rpl37a, Rpl38, Rpl39, Rps14, Rps15a, Rps21, Rps24, Rps27), and translation initiation factor eIF2α genes (Eif2s1, Eif2s2) were upregulated in *Mpl^–/–^ Dnm2^Plt–/–^* mice. The observed increase in erythroferrone (Erfe) is consistent with severe anemia and grossly elevated plasma EPO levels (63).

## Discussion

Here, we assessed whether the hyperproliferative phenotype of *Dnm2^Plt–/–^* mice specifically lacking DNM2 in platelets and MKs was due to JAK2 constitutive activation or elevated circulating TPO levels (42). Our data shows that DNM2-dependent Mpl-mediated endocytosis in the MK/platelet lineage is required for steady-state hematopoiesis and provides novel insights into a developmentally controlled role for Mpl in normal erythropoiesis.

STAT3 and, to a lower extent, STAT5 were tyrosine phosphorylated in *Dnm2^Plt–/–^* platelets in the absence of TPO, consistent with JAK2 constitutive activation (42). However, STAT3 and STAT5 phosphorylation were slowed and diminished following stimulation with TPO. The slowing of STAT phosphorylation indicated that the MK hyperplasia of *Dnm2^Plt–/–^* mice was not due to constitutive Mpl signaling in MKs. Instead, the phenotype of *Dnm2^Plt–/–^* mice resembles that of mice lacking Mpl or JAK2 in platelets and MKs, in which Mpl-mediated JAK-STAT signaling and TPO endocytosis are blunted (33–35). To ascertain whether elevated plasma TPO levels stimulating Mpl-expressing HSCs led to the severe MK hyperplasia and HSPC expansion, we generated *Mpl^–/–^ Dnm2^Plt–/–^* mice lacking DNM2 in platelets and MKs and Mpl ubiquitously. JAK2 expression was further diminished in *Mpl^–/–^ Dnm2^Plt–/–^* platelets. STAT3, but not STAT5 expression was also decreased, suggesting that JAK2 and STAT3 expression in platelets and MKs are intimately regulated by DNM2 and Mpl. JAK2 expression was also decreased in *Mpl^–/–^* platelets, consistent with a scaffolding role for Mpl (64–66). Further proteomics and RNAseq analysis data may reveal how Mpl and DNM2 regulate the expression of these and other platelet and MK proteins.

Ubiquitous Mpl deletion in *Mpl^–/–^ Dnm2^Plt–/–^* mice resulted in a severe deficiency of bone marrow MKs and HSCs, like in *Mpl^–/–^* mice, demonstrating that impaired Mpl-mediated endocytosis in platelets and MKs lacking DNM2 is responsible for the MK hyperplasia and HSC expansion of *Dnm2^Plt–/–^* mice. Ablation of the MK population and HSC niche in *Mpl^–/–^* mice is consistent with previous studies characterizing the mouse model (14, 15, 67), and others using a signaling deficient, cell surface truncated form of the receptor (68, 69). Mpl deficiency is associated with abnormal maturation of neonatal MKs and developmental stage-specific defects in platelet function (44). Hence the MK defect and thrombocytopenia result from defective and inefficient MKs. Comparing *Mpl^–/–^* and *Thpo^–/–^* mice revealed that Mpl expression, but not TPO, was critical for the hyperproliferative phenotype of a JAK2V617F+ MPN mouse model, including the splenomegaly (66). The authors hypothesized that expression of hyperactive JAK2V617F in HSCs was likely decreased in the absence of its chaperone, Mpl. In our experiments, Mpl deletion did not alleviate the severe splenomegaly of *Mpl^–/–^ Dnm2^Plt–/–^* mice, indicating that other mechanisms are at play. The data suggests that extramedullary hematopoiesis, including the production of premature erythrocytes, occurs in the spleen despite the loss of DNM2-dependent endocytosis in platelets and MKs and Mpl deletion. However, the precise role of DNM2 and Mpl in regulating spleen homeostasis remains to be determined.

We did not anticipate the premature death of *Mpl^–/–^ Dnm2^Plt–/–^* mice at P25, which was attributed to severe anemia and disrupted EB maturation in early development. Increased circulating reticulated RBCs and grossly elevated plasma EPO levels confirmed a rise in stress erythropoiesis. *Dnm2* was not excised in EBs isolated from *Dnm2^Plt–/–^* mice, confirming that DNM2 was normally expressed in erythroid progenitors. The data, therefore, excludes defective DNM2-dependent CD71-mediated transferrin uptake in EBs as the cause of the severe anemia, as has been described in mice expressing the *Dnm2* loss-of-function mutation V235G ubiquitously (41). This poses the question of how additional Mpl deletion in the platelet- and MK-specific *Dnm2^Plt–/–^* background aggravates the RBC phenotype.

Our RNA sequencing analysis supports the notion that Mpl plays a critical role in regulating hemoglobin and heme homeostasis in EBs, as associated genes (Hbb-bs, Hbb-bt, Hbq1a, Alas2, Fech, Bpgm, Ftl1, Slc48a1) were significantly decreased in EBs lacking Mpl, independently of DNM2 deletion in platelets and MKs. Additional downregulated genes are involved in ubiquitination (Ubb, Mkrn1, Marchf2, Usp15) and ribosome recruitment and translation initiation (Pabpc1). Hemoglobin production depends on the fine-tuned sequential process of joining two α-globin and two β-globin subunits with the addition of attached iron-binding heme groups. In β-thalassemia, overabundant α-globin is polyubiquitinated and targeted for protein degradation, thereby preventing proteotoxicity (70, 71). The data further suggests poor proteome integrity in EBs lacking Mpl, as the RNA-binding E3 ubiquitin ligase Makorin 1 (Mkrn1) interacts with poly(A)-binding protein 1 (Pabpc1) to maintain ribosome-associated quality control of poly(A) translation (72). Alternatively, H2A cluster histone genes (H2ac7, H2ac10, H2ac12, H2ac13, H2ac14) were upregulated in EBs lacking Mpl. Whether this increase contributes to chromatin condensation and enucleation required for RBC formation remains to be determined (73, 74).

Remarkably, DNM2 deletion in platelets and MKs resulted in upregulation of E2 ubiquitin-conjugating enzymes (Ube2l3, Ube2n) and cytochrome c oxidase (Cox5b, Cox7c) in EBs, independently of Mpl ubiquitous deletion. Ube2l3 and Ube2n have been implicated in autophagic clearance of depolarized mitochondria (75), suggesting increased mitophagy in EBs in mice lacking DNM2 in platelets and MKs. E3 ubiquitin ligase genes (Cish, Rbx1), genes involved in ribosome biogenesis (Rpl21, Rpl22l1, Rpl23, Rpl27a, Rpl28, Rpl35, Rpl36, Rpl37, Rpl37a, Rpl38, Rpl39, Rps14, Rps15a, Rps21, Rps24, Rps27), and translation initiation factor eIF2α genes (Eif2s1, Eif2s2) were upregulated in *Mpl^–/–^ Dnm2^Plt–/–^* mice, consistent with increased protein translation (76, 77). Together, the data suggests that the severe anemia and early mortality of *Mpl^–/–^ Dnm2^Plt–/–^* mice is due to the combined effects of decreased hemoglobin and heme production, mitochondrial dysfunction, and proteotoxicity, resulting from increased, but poorly quality-controlled protein translation in EBs.

How does specific DNM2 deletion in the MK/platelet lineage affect the expression of these genes and ultimately erythroid development? One possibility is that MKs and platelets internalize cytokines to contribute to regulating EB maturation. In the absence of DNM2-dependent endocytosis in the MK/platelet lineage, combined with the severe MK hypoplasia and thrombocytopenia in the *Mpl^–/–^* background, increased levels of these cytokines lead to EB maturation blockage. MKs are the primary source of transforming growth factor β1 (TGF-β1) and as such regulate steady-state erythropoiesis by restraining progenitor cell and EB production (78). MK TGF-β1 and platelet factor 4 (PF4) also maintains HSC quiescence during homeostasis and promotes HSC regeneration after chemotherapeutic stress (79, 80). A second hypothesis is that Mpl deficiency greatly limits the proliferation of the bone marrow HSPC pool, thereby reducing the availability of differentiating HSCs to develop along the erythroid lineage. The consequences of Mpl loss on erythroid development have been reported previously in studies using induced pluripotent stem cells (iPSCs) derived from CAMT patients, where deficient Mpl signaling results in a loss of MEP differentiation and is critical for successful erythropoiesis (81).

While their circulating RBC and reticulocyte counts were normal at P24 and P56, *Mpl^–/–^ Dnm2^Plt+/+^* mice developed a transient erythropoiesis defect, with a percentage of mature EBs about half that of *Dnm2^Plt+/+^* mice, which was only apparent during early development (P24). By adulthood (P56), *Mpl^–/–^ Dnm2^Plt+/+^* mice were able to normalize the EB maturation defect. The data indicates that Mpl regulates erythropoiesis during early development in mice. TPO expands erythroid progenitors, increases RBC production, and enhances erythroid recovery following myelosuppressive therapy (82). Others have implicated an EPO-independent, macrophage-associated pathway supporting terminal erythropoiesis in this expansion system in humans (83). The fetal/neonatal hematopoietic system must generate enough blood cells to meet the demands of rapid growth. This unique challenge might underlie the high incidence of thrombocytopenia among preterm neonates (44). It is possible that under developmental stress and increased need for blood production, there is crosstalk between EPO and TPO signaling in regulating hematopoiesis to produce platelets and RBCs efficiently simultaneously. Therefore, Mpl-dependent erythropoiesis is likely more significant than expected under pathological and developmental pressure to produce platelets and RBCs rapidly. Conversely, EPO can also induce megakaryopoiesis, supporting the notion that the two pathways cooperate to ensure platelet and RBC numbers (84).

In conclusion, DNM2-dependent Mpl-mediated endocytosis in platelets and MKs is required for steady-state hematopoiesis. It provides novel insights into a developmentally controlled role for Mpl in normal erythropoiesis, regulating hemoglobin and heme production.

## Data availability statement

The original contributions presented in the study are publicly available. This data can be found in the NCBI GEO repository under accession number GSE206343.

## Ethics statement

The animal study was reviewed and approved by Medical College of Wisconsin Institutional Animal Care and Use Committee guidelines (Animal Use Application 5600).

## Author contributions

NE, EB, RB, ML-S, TD, and HR designed and performed experiments, collected, analyzed, and interpreted data, and revised the manuscript. SZ and RB analyzed the RNAseq data. MS-V and KH analyzed and interpreted data and revised the manuscript. HF conceived and designed the study, designed and performed experiments, collected, analyzed, and interpreted data, and wrote and revised the manuscript. All authors contributed to the article and approved the submitted version.

## Funding

This work was supported by the American Society of Hematology Foundation (H.F.) and National Institutes of Health, National Heart, Lung, and Blood Institute grants HL046925 (M.C.S.V.), HL089224, HL141954, HL151333 (K.M.H.), and HL126743 (H.F.).

## Acknowledgments

We thank Jon Wieser for technical assistance and Drs Hartmut Weiler and Anthony Veltri for helpful discussion. 

## Conflict of interest

The authors declare that the research was conducted in the absence of any commercial or financial relationships that could be construed as a potential conflict of interest.

## Publisher’s note

All claims expressed in this article are solely those of the authors and do not necessarily represent those of their affiliated organizations, or those of the publisher, the editors and the reviewers. Any product that may be evaluated in this article, or claim that may be made by its manufacturer, is not guaranteed or endorsed by the publisher.

## Supplementary material

Supplementary Figure 1 The MK hyperplasia of *Dnm2^Plt–/–^* mice requires Mpl expression. H&E staining of femur bone marrow **(A)** and spleen **(B)** sections, and thin blood smears **(C)** of *Dnm2^Plt+/+^*, *Dnm2^Plt–/–^*, *Mpl^–/–^ Dnm2^Plt+/+^*, and *Mpl^–/–^ Dnm2^Plt–/–^* mice at P24. Data shown are representative of 3 mice in each genotype. Scale bars represent 40 μm.
