# Supplementary figures and images for: Endocytosis of the thrombopoietin receptor Mpl regulates megakaryocyte and erythroid maturation in mice
Source: Front Oncol. 2022 Aug 30;12:959806. doi: 10.3389/fonc.2022.959806 (PMC9468709; doi:10.3389/fonc.2022.959806)

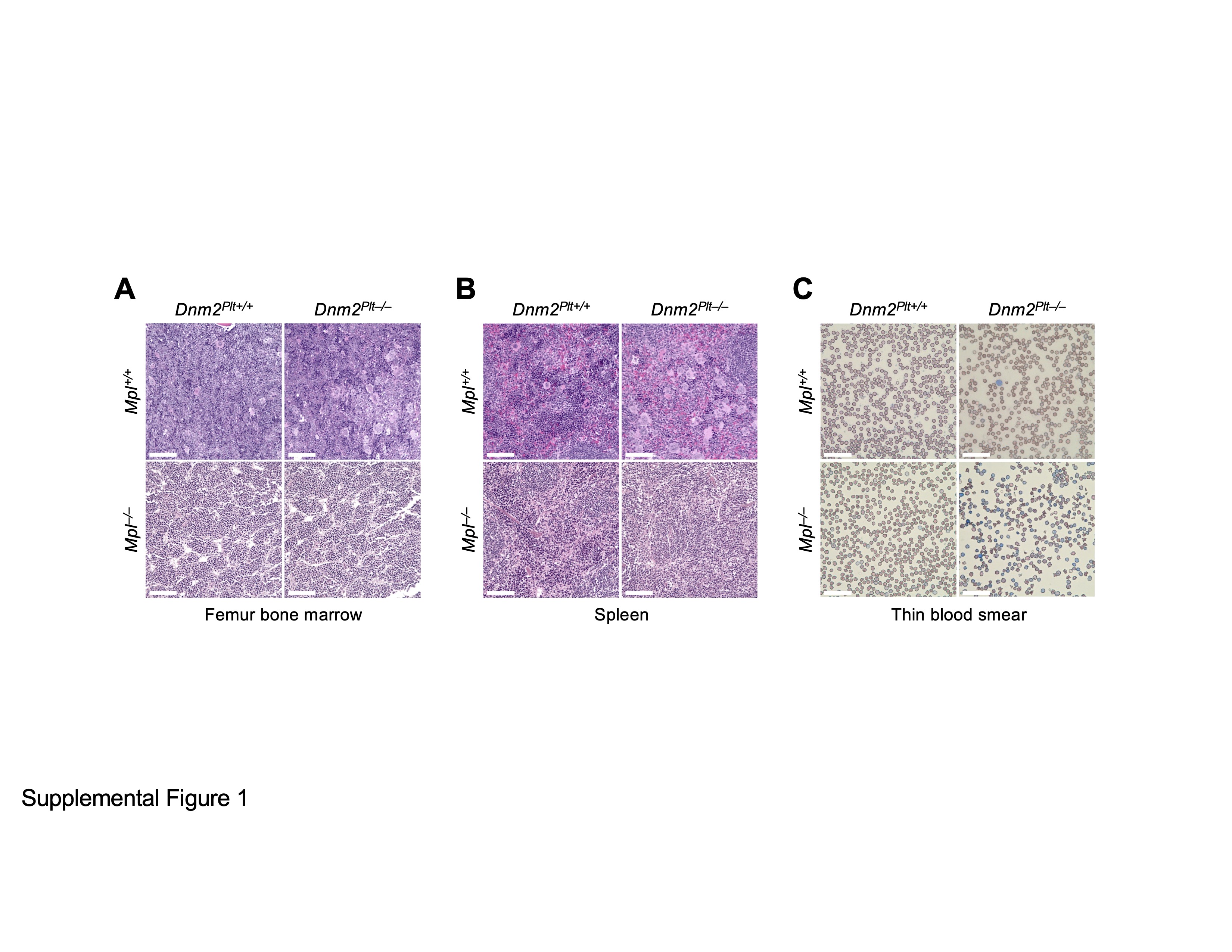

Supplement: Supplementary Figure 1 — The MK hyperplasia of Dnm2Plt–/– mice requires Mpl expression. H&E staining of femur bone marrow (A) and spleen (B) sections, and thin blood smears (C) of Dnm2Plt+/+ , Dnm2Plt–/– , Mpl–/– Dnm2Plt+/+ , and Mpl–/– Dnm2Plt–/– mice at P24. Data shown are representative of 3 mice in each genotype. Scale bars represent 40 μm. [file Image_1.jpeg]
